# Antidepressant-like Effects of δ Opioid Receptor Agonists in Animal Models

**DOI:** 10.2174/157015912803217314

**Published:** 2012-09

**Authors:** Akiyoshi Saitoh, Mitsuhiko Yamada

**Affiliations:** Department of Neuropsychopharmacology, National Institute of Mental Health, National Center of Neurology and Psychiatry, Tokyo 187-8553, Japan

**Keywords:** Anxiety, depression, KNT-127, naltrindole, SNC80, TAN-67.

## Abstract

Recently, δ opioid receptor agonists have been proposed to be attractive targets for the development of novel antidepressants. Several studies revealed that single treatment of δ opioid receptor agonists produce antidepressant-like effects in the forced swimming test, which is one of the most popular animal models for screening antidepressants. In addition, subchronic treatment with δ opioid receptor agonists has been shown to completely attenuate the hyperemotional responses found in olfactory bulbectomized rats. This animal model exhibits hyperemotional behavior that may mimic the anxiety, aggression, and irritability found in depressed patients, suggesting that δ opioid receptor agonists could be effective in the treatment of these symptoms in depression. On the other hand, prototype δ opioid receptor agonists produce convulsive effects, which limit their therapeutic potential and clinical development. In this review, we presented the current knowledge regarding the antidepressant-like effects of δ opioid receptor agonists, which include some recently developed drugs lacking convulsive effects.

## 1 INTRODUCTION

Depression is one of the major psychiatric diseases. It is estimated to affect 13–17% of the population at some point during the lifetime of an individual [1, 2]. All antidepressants have been developed based on the monoaminergic deficit hypothesis of depression, which was proposed in the 1950s. These antidepressants immediately increase synaptic concentrations of norepinephrine and/or serotonin. However, in general, a latency period of several weeks elapses before the therapeutic effects of anti-depressants are observed. This discrepancy implies that mechanisms beyond monoaminergic systems are involved in the treatment of depression. Importantly, fewer than 50% of all patients with depression show full remission with optimized treatment, including trials on numerous medications with and without concurrent psychotherapy [3]. Therefore, there remains a great need for the development of faster-acting, safer, and more effective compounds for depression treatment.

Recently, δ opioid receptor agonists have been proposed to be attractive targets for the development of novel antidepressants [4-6]. There are, however, the preclinical studies that a portion of non-peptidic δ opioid receptor agonists produced convulsive effects (see review by Pradhan *et al*. (2011) [7]). As a result, clinical development of δ opioid receptor agonists has been limited. In this review, we presented the current knowledge regarding the antidepressant-like effects of δ opioid receptor agonists, which include some recently developed drugs lacking convulsive effects.

## 2 ENDOGENOUS OPIOID SYSTEMS AND DEPRESSION

The biological effects of endogenous opioid peptides are mediated through three classes of naloxone-sensitive opioid receptors: mu (µ), kappa (κ), and delta (δ) (Table **1**). Several lines of evidence have implicated a role for opioid receptor systems in depression, including early studies investigating the potential antidepressant therapy of endogenous opioid peptides in humans. For example, it was shown that basal serum β-endorphin (an endogenous opioid peptide that binds to µ and δ opioid receptors) levels were significantly elevated in patients with depression after antidepressant treatment [8, 9]. It was also reported that plasma β-endorphins levels were found to be elevated in patients after receiving electroconvulsive shock (ECS) for the treatment of depression, suggesting endogenous µ and/or δ opioid receptors were involved, at least in part, in the mechanisms of the antidepressant activity of ECS [10, 11]. In fact, it was reported that β-endorphin rapidly produced an antidepressant action in depressed patients [12]. Some clinical reports also demonstrated the effectiveness of the µ opioid receptor agonists oxycodone, oxymorphone and buprenorphine in patients with refractory major depression [13, 14]. Conversely, it was indicated that the non-selective opioid receptor antagonist naltrexone induced self-reported mental depression to volunteer subjects in a placebo-controlled open study [15]. Taken together, these findings suggest that endogenous opioid systems have important roles in depression.

The δ opioid receptor was cloned in the 1990s [16, 17]. It was reported that these δ opioid receptors were located in the olfactory bulb, cerebral cortex, striatum, amygdala, hippocampus, brainstem nuclei and spinal cord in rodents [7, 18]. Madar *et al.* (1996) suggest that the distribution pattern of δ opioid receptors in the human brain using [11C]-methyl-naltrindole was partially consistent with the location of the major regions involved in the modulation of mood and emotion (Table **2**) [19]. Interestingly, Filliol *et al*. (2000) found that δ opioid receptor knockout mice exhibited increases in the immobility time in the forced swimming test. This finding also suggests that the endogenous δ opioid receptor systems significantly contribute to the regulation of mood and emotion.

## 3 ANTIDEPRESSANT-LIKE EFFECTS OF δ OPIOID RECEPTOR AGONISTS IN ANIMAL MODELS

### 3.1 Antidepressant-like Effects by Single Treatment

The antidepressant potential of non-peptidic δ opioid receptor agonists has mainly been evaluated in the forced swimming test (Table **3**). The forced swimming test was developed by Porsolt *et al*. (1977) [20]. It has become the most popular test in animals to evaluate antidepressant-like potential. The widespread use of this test is largely a result of its ease of use, reliability between research centers, and ability to detect a wide range of antidepressant-like activities [21, 22]. Cryan *et al*. (2005) recently developed the modified Porsolt forced swimming test, which had good sensitivity to detect the effects of antidepressants in rodents [22]. This test involves measuring the immobility, swimming behavior and climbing behavior of rodents upon subsequent exposure to swimming [23, 24]. All currently approved therapeutic antidepressant compounds are active in this test by decreasing immobility scores compared with vehicle controls. For example, results demonstrated that the analysis of antidepressant drugs in the forced swimming test allow discrimination between serotonergic drugs (e.g., fluoxetine) that reduce immobility by increasing swimming, and noradrenergic (e.g., desipramine) and dopaminergic (e.g., buproprion) drugs that reduce immobility by increasing climbing [25, 26]. Other antidepressant drugs such as the tricyclic antidepressants imipramine and the dual noradrenalin/serotonin reuptake inhibitor milnacipran dose-dependently reduce immobility time, and equally increase climbing and swimming behaviors [27, 28]. Therefore, the forced swimming test should reflect the predictive validity to screen for compounds with potential antidepressant activity.

Nagase *et al*. (2002) found that subcutaneous administration of the non-peptidic selective δ opioid receptor agonist TAN-67 [29, 30] at the dose of 1–10 mg/kg in rats produced significant increases in the duration of swimming in the forced swimming test after subcutaneous administration [29]. These findings were similarly demonstrated in studies using other non-peptidic selective δ opioid receptor agonists. It was reported that SNC80 and its derivate (+) BW373U86 produced antidepressant-like effects in the forced swimming test of rats [23] and mice [30], as evidenced by a decrease in the duration of immobility and increase in the duration of climbing and swimming. In addition, the δ opioid receptor antagonist naltrindole prevented the decrease in the immobility and increase in climbing activity seen with the administration of SNC80 and (+) BW373U86. These results suggested that SNC80 and (+) BW373U86 showed δ opioid receptor-mediated antidepressant-like effects in the forced swimming test. Interestingly, the non-peptidic selective κ opioid receptor agonist CI-977 (1–3.2 mg/kg) and the µ opioid receptor agonist morphine (1–10 mg/kg) did not significantly affect the duration of immobility and swimming, but significantly decreased the duration of climbing, after subcutaneous administration. These results indicated that activation of δ (rather than µ or κ) opioid receptors are needed to produce antidepressant-like effects in the forced swimming test.

Recently, Nagase *et al*. (2010) succeeded in synthesizing a novel non-peptidic δ opioid receptor agonist called KNT-127 [31]. KNT-127 was designed based on the structure of TAN-67 and its derivative SN-28 [32]. KNT-127 showed higher affinity for δ opioid receptors (dissociation constant (K_i_) = 0.16 nM) than TAN-67 and lower affinity for µ opioid receptors (K_i _= 21.3 nM) and κ opioid receptors (K_i_ = 153 nM). Consequently, KNT-127 showed higher selectivity for δ opioid receptors than SN-28, suggesting the sufficient affinity and selectivity for δ opioid receptors [31]. Interestingly, subcutaneous administration of KNT-127 (0.1–1 mg/kg), produced the significant decreases in the duration of immobility and the significant increases in the duration of swimming in the forced swimming test, without influencing spontaneous locomotor activity in mice. In addition, these behavioral changes induced by KNT-127 (1 mg/kg) were completely antagonized by the selective δ opioid receptor antagonist naltrindole and were similar to that observed for the tricyclic antidepressant imipramine (6 mg/kg).

In addition to the results of the forced swimming test, it was shown that subcutaneous administration of SNC80 (1–3 mg/kg) completely reversed the conditioned suppression of motility in the conditioned fear stress model, reflecting psychological stress-induced motor suppression [30]. These effects were antagonized by the selective δ-opioid-receptor antagonist naltrindole (1 mg/kg). It was known that the conditioned suppression of motility in the conditioned fear stress model is attenuated by antidepressants such as SSRIs as well as anxiolytics [33-35]. And also, this model was known as the animal model to examine emotional states such as motivation and/or anxiety [36, 37], and was proposed as an animal model that partially reflected the construct validity of depression.

Recently, other novel δ opioid receptor agonists were developed and proposed as candidate compounds for anti-depressants. Naidu *et al*. (2007) demonstrated that NIH11082 produced δ opioid receptor-mediated antidepressant-like effects in the mouse tail suppression test (a test used to screen SSRI antidepressant agents) [38]. Vergura *et al*. (2008) reported that the peptidic δ opioid receptor agonist UFP-512 significantly reduced immobility time during intracerebroventricular (0.01 nmol) and intraperitoneal (0.1–0.3 mg/kg) administration in the forced swimming test in mice [39]. Recently, it was reported that ADL5859 (3 mg/kg, p.o.) produced antidepressant-like activity, as evidenced by a significant decrease in the duration of immobility and a significant increase in the duration of swimming in the forced swimming test [40]. Oral administration of ADL5859 (1–10 mg/kg) also attenuated the number of escape failures after inescapable shock in the learned helplessness test in rats, reflecting predictive validity of depression, identical to the effects of imipramine (20 mg/kg). More recently, it was reported that oral administration of AZD2327 (1 mg/kg) produced the antidepressant-like effects in the learned helplessness test in rats [41]. These results reinforce the notion that δ opioid receptor agonists can produce significant antidepressant-like effects in behavioral models.

Thus, these results mediated by δ opioid receptors in several animal models reflected predictive and construct validity of depression strongly suggest that δ opioid receptor agonists could improve the depressed state.

### 3.2 Antidepressant-like Effects by Subchronic Treatment 

The pharmacological effects of monoaminergic reuptake inhibitors are produced within a few hours after antidepressant treatment. The forced swimming test and tail suspension test are used to evaluate the activity of antidepressants within a few hours after antidepressant treatment in rodents. However, in general, a latency period of several weeks elapses before the therapeutic effects of antidepressants are observed in patients. Interestingly, the olfactory bulbectomized (OB) rat model is unique in that it is used to evaluate the activity of antidepressants after chronic antidepressant treatment [42].

Olfactory bulbectomy results in a complex constellation of behavioral, neurochemical, neuroendocrine, and neuro-immune alterations, many of which reflect symptoms reported in patients with major depression [42]. These changes typically require 1–2 weeks to develop and occur independent of sensory deprivation [42, 43]. Several investigators have demonstrated that the behavioral effects of olfactory bulbectomy induce increases in various measures of locomotor activity, irritability and hyperemotionality [44, 45]. It has been suggested that such behaviors resemble psychomotor agitation, which is a diagnostic criterion for depression. In addition to behavioral alterations, olfactory bulbectomy has been reported to alter adrenergic, serotonergic, and excitatory amino-acid receptors in cortical and limbic areas in a fashion similar to the receptor alterations observed in suicide victims, and immune functions were suppressed in a manner consistent with the immune suppression seen in clinical depression [42, 43]. In addition, it was reported that olfactory bulbectomy resulted in a significant reduction in neurogenesis in the hippocampal and subventricular zone [46, 47]. Importantly, these behavioral, neurochemical and immune alterations in OB rats could be improved by subchronic treatment with monoaminergic antidepressants [42, 43]. Therefore, it has been proposed that OB rats were useful models of depression to screen candidate therapeutic compounds [48, 49, 50]. Based on these findings, the OB rat model is considered to be one of the important animal models of depression in terms of face and predictive validity [42, 43].

Previously, we found that the hyperemotional behaviors, such as bite, startle, struggle, and fight responses in OB rats were significantly reversed after oral subchronic treatment for 7 days with the serotonin noradrenalin reuptake inhibitor (SNRI) milnacipran (3–10 mg/kg) and/or the SSRI fluvoxamine (10–30 mg/kg) [51]. Interestingly, single treatment with neither milnacipran nor fluvoxamine showed significant effects in the hyperemotional behaviors of OB rats. Subsequently, we examined antidepressant-like effects in OB rats by subchronic treatment of the δ opioid receptor agonist SNC80 [52]. SNC80 (1–10 mg/kg, i.p.) showed dose-dependent and time-dependent decreases in the hyperemotional responses of OB rats on day 3, 5 and 7 [52]. Interestingly, these effects of SNC80 were observed in OB rats at least 24 hours after final administration on day 7 [53]. These significant improvements in the hyperemotionality by SNC80 (1 and 10 mg/kg) were significantly greater than those by the tricyclic antidepressant desipramine (10 mg/kg, i.p.). Surprisingly, SNC80 completely reversed the emotional scores to the same levels found in sham-operated control rats. In addition, SNC80 produced the significant inhibition of hyperemotional behaviors of OB rats even more on day 7 compared with those on day 3 and 5, suggesting that the antidepressant-like effect of SNC80 in OB rats does not cause tolerance. OB models may be useful for predicting the antidepressant action of δ opioid receptor agonists after chronic administration.

The antidepressant-like effect of the δ opioid receptor agonist was mainly examined in the forced swimming test using SD rat strain. The acute treatment with the δ opioid receptor agonist (+) BW373U86 produced the antidepressant-like effects [4], while the subchronic treatment led to the development of tolerance to the antidepressant-like effects in the forced swimming test using SD rat strain [54]. In contrast, Wistar rats were used in the OB models. These different strains may be involved in the different reaction to chronic treatment paradigms.

The hyperemotional responses of the OB model may imitate the psychiatric symptoms (e.g., psychomotor agitation, anxiety, aggression, and irritability) found in depressed patients. Our results suggest that a δ opioid receptor agonist could rapidly and effectively treat these symptoms in depression. Indeed, AZD2327 is being assessed in a Phase II trial in adult patients with anxious major depressive disorder (clinicaltrials.gov ID NCT00759395).

## 4 MECHANISM OF ANTIDEPRESSANT-LIKE EFFECTS BY δ OPIOID RECEPTOR AGONISTS

The mechanism responsible for the antidepressant-like effects induced by δ opioid receptor agonists remains unknown. However, it has been suggested that dysfunction of the serotonergic system could be reversed by activation of δ opioid receptors. Several authors have stated that the tissue concentration of serotonin and its metabolite 5-Hydroxyindole acetic acid (5-HIAA) in OB rats are decreased in the mid-brain and limbic-cortical areas, such as frontal cortex, hippocampus and amygdala (see review by Song and Leonard (2005) [43]). It is widely accepted that these limbic-cortical areas (which contain the main projections of serotonergic neurons from the dorsal raphe nucleus) are important for regulating mood and anxiety. In this regard, it was demonstrated that these changes in OB rats were reversed after chronic treatment with SSRIs, SNRIs, and tricyclic antidepressants (see review by Song and Leonard (2005) [43]). We also provided the first histological evidence that olfactory bulbectomy significantly decreased levels of tryptophan hydroxylase (TPH; which is the rate-limiting enzyme of serotonin synthesis) in the dorsal raphe compared with sham-operated rats, and that these losses of TPH-positive cells in OB rats were dramatically reversed by subchronic treatment with the SSRI fluvoxamine and the SNRI milnacipran [51]. These findings were in accordance with autoradiographic studies stating that the rate of serotonin synthesis was decreased in the dorsal raphe of OB rats [55], and that these decreases were reversed by subchronic treatment with the SSRI citalopram [56].

Previously, we have demonstrated that significant decreased in the tissue concentration of serotonin and 5-HIAA in the frontal cortex, hippocampus, and amygdala were normalized by subchronic treatment of δ opioid receptor agonist SNC80 in OB rats. SNC80 also significantly reversed the loss of TPH-positive cells produced by olfactory bulbectomy in the dorsal raphe [52]. Furthermore, it was recently reported that non-peptidic δ opioid receptor antagonists such as NTI and its derivatives (HS-378, HS-459) suppressed the degradation of the serotonin precursor tryptophan [57], suggesting that δ opioid receptors could modulate the availability of tryptophan and serotonin. These results suggest that the repair of serotonergic abnormalities observed by subchronic treatment with SNC80 may play a key part in improving hyperemotionality and anxiety-like behavior in the elevated plus maze test of OB rats.

Conversely, it was reported that peptidic and non-peptidic δ opioid receptor agonists increase brain-derived neurotrophic factor (BDNF) gene expression across several brain regions [54, 58, 59]. Interestingly, when (+) BW373U86 (1–10 mg/kg) was acutely administrated in rats, expression of BDNF mRNA was significantly increased in the frontal cortex, hippocampus, basolateral amygdala, endopiriform nucleus, and primary olfactory cortex. Siuciak *et al*. (1998) reported that chronic BDNF treatment induced the increase in the tryptophan hydroxylase mRNA levels and the 5-HT contents in the rat raphe uncles [60]. Furthermore, it was suggested that stimulation of the δ opioid receptor may mediate neurogenesis and neuroprotection through BDNF release [61]. Although further investigation is required, the improvement of serotonergic abnormalities in the limbic cortical area by chronic treatment with SNC80 may be involved with δ opioid receptor-induced BDNF changes.

Although only one δ opioid receptor gene has been cloned [16, 17], *in vivo* pharmacological studies suggest that at least two subtypes of the δ opioid receptor are expressed: the putative δ_1_ opioid receptor subtype and the putative δ_2_ opioid receptor subtype. Interestingly, it was reported that the antidepressant-like effects of KNT-127 in mice were antagonized by pretreatment with the putative δ_2_ opioid receptor antagonist [D-Ala^2^, D-Glu^4^] deltorphin and blocked by naltriben (NTB) but not by the putative δ_1_ opioid receptor antagonist 7-benzylidenenaltrexone (BNTX) [62]. These results suggest that the putative δ_2_ opioid receptor subtype could play an important part in regulating depressive-like behaviors in rodents.

## 5 CONVULSIVE EFFECTS OF δ OPIOID RECEPTOR AGONISTS

**Fig. (1):**
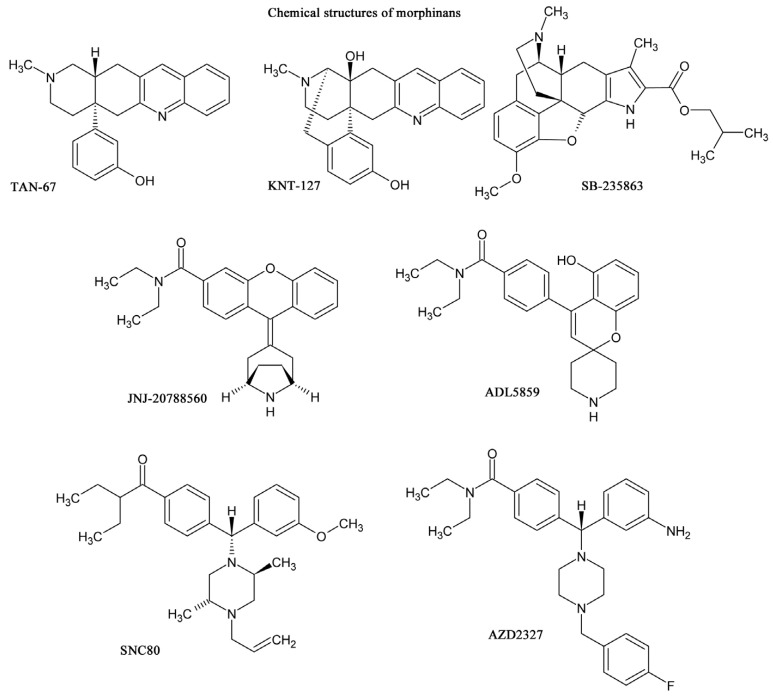
Chemical structures of representative δ opioid receptor agonists

In general, peptide δ opioid receptor agonists do not produce convulsions in rodents [39, 63]. However, it was reported by Comer and colleagues (1993) that the non-peptidic δ opioid receptor agonist (+) BW373U86 has convulsive activity [64]. They reported that after systemic injection, (+) BW373U86 caused dose-dependent convulsive activity in mice. However, unlike the convulsant agent pentylenetetrazol, (+) BW373U86 afforded only a single convulsive episode that was not lethal at doses that induced a convulsion in all mice [64]. The convulsion induced by non-peptidic δ opioid receptor agonists such as SNC80 and (+) BW373U86 are characteristically brief (20–30 s), non-lethal, and occur from approximately 4–15 min after systemic administration. A short period (5–10 min) of catalepsy is seen after the convulsive episode, after which the animals appear to make a rapid recovery. These effects were antagonized by δ opioid receptor antagonist naltrindole, nonselective opioid receptor antagonist naltrexone and benzodiazepine/GABA_A_ receptor agonist midazolam [4]. Moreover, the lack of SNC80-induced convulsions in δ opioid receptor knockout mice suggested that δ opioid receptor and GABAergic systems are required for this effect. Importantly, Broom *et al*., (2002) reported that the convulsive effects of non-peptidic δ opioid receptor agonists were independent of antidepressant-like effects in rats because the convulsive effects of the non-peptidic δ opioid receptor agonist (+) BW373U86 were blocked by midazolam without altering its antidepressant-like effects [65]. Indeed, the convulsive effects were not required for its antidepressant-like effects. These results suggested the possibility of discovering δ opioid receptor agonists that maintain their antidepressant profile without adverse convulsive effects. As supported these notion, recent studies on δ opioid receptor agonists (Fig. **1**), such as (–)–NIH 11082 [64], SB-235863 [67], ADL5859 [40], ADL5747 [68], JNJ-20788560 [69] and AZD2327 [41], KNT-127 [62], have failed to demonstrate convulsive effects with these agonists. We demonstrated that KNT-127 produced no convulsions or catalepsy, even at a much higher dose (100 mg/kg) than those required for antidepressant-like and antinociceptive effects in mice [62].

The detailed mechanisms of convulsive effects by δ opioid receptor agonists are unknown. Interestingly, both non-convulsive δ opioid receptor agonist KNT-127 [62] and SB-235863 [67] has a characteristic morphinan chemical structure (Fig. **1**), which was designed according to an extension of the message–address concept proposed by Portoghese (1989) [70]. In this regard, pro-convulsive δ opioid receptor agonist SNC80 and related compounds are commonly classified as having a diarylpiperidine chemical structure. Pro-convulsant activity produced by some of the δ opioid receptor agonist may be dependent upon the features of the structure and not of δ opioid receptor agonists *per se*. In addition, Pradhan *et al*., (2009) recently reported that several types of internalization processes of δ opioid receptors are present within several δ opioid receptor agonists, and proposed that these different processes may underlie differential potency and efficacy of binding [71]. These different internalization processes of the δ opioid receptor may be related to the pharmacological profiles of δ opioid receptor agonists.

## 6 CONCLUSION

Various animal models have been used for pharmacological studies of δ opioid receptor agonists. These agonists are attracting attention as a new potential treatment for depression. So far, adverse effects such as convulsions have limited their therapeutic potential and clinical development. Therefore, δ opioid receptor agonists without adverse effects (e.g., KNT-127 and its derivates) should be considered as candidate compounds for the development of antidepressants with a novel class of action.

## Figures and Tables

**Table 1.** Pharmacology of µ-, κ- and δ-Opioid Receptors

Type	µ	κ	δ

**Endogenous ligand**	Endomorphin	Dynorphin	Enkephalin

**Agonist**	Morphine*	Nalfurafine*	SNC80
	Fentanyl*	Pentazocine*	KNT-127
			AZD2327#

**Antagonist**	β-FNA	nor-BNI	Naltrindole
	Naloxonazine		Naltriben
	Naloxone*		BNTX

**Effects**	Antinociceptive	Antipruritic	Antidepressant
	Antitussive	Antinociceptive	Anxiolytic
	Gastrointestinal inhibition	Antidepressant	Antinociceptive
	Respiratory inhibition	Diuresis	Antitussive
	Reward	Sedative	Urinary incontinence treatment

* Clinically approved drug

# Phase II trial, β-Funaltrexamine (β-FNA), Norbinaltorphimine (nor-BNI), 7-Benzylidenenaltrexone (BNTX).

**Table 2.** The Distribution of δ Opioid Receptor in Human Brain

Density	Region
**High**	Neocortex (insular, occipital, frontal, parietal, cingulated, temporal) Putamen, Caudate
**Intermediate**	Amygdala
**Low**	Cerebellum Thalamus

The distribution pattern was referred to the reports by Madar *et al*. (1996) in human brain PET studies, using [^11^C]-methyl-naltrindole.

**Table 3.** Antidepressant-Like Effects of δ Opioid Receptor Agonists in Animal Models

#	Author	Year	Compound	Strain	Dose	Rote	Test	(Model)
1	Nagase *et al*.	2002	TAN-67	SD	1-10 mg/kg	s.c.	FSS	
2	Broom *et al*.	2002	SNC80	SD	10-100 mg/kg	s.c.	FSS	
3	Broom *et al*.	2002	(+) BW373U86	SD	1-10 mg/kg	s.c.	FSS	
4	Saitoh *et al*.	2004	SNC80	ICR	0.3-3 mg/kg	s.c.	FSS/CSM	
5	Torregrossa *et al*.	2005	(+) BW373U86	SD	10 mg/kg X 1, 8, 21 days	s.c.	FSS	
6	Naidu *et al*.	2007	(-) NIH11082	C57BL6	8-32 mg/kg	i.p.	TST	
7	Saitoh *et al*.	2008	SNC80	Wistar	1-10 mg/kg X 7 days	s.c.	HE	OB rat model
8	Vergra *et al*.	2008	UFP-512	Swiss	0.001-0.1 nmol/0.01-1 mg/kg	i.c.v./i.p.	FSS	
9			UFP-512	SD	0.1-1 mg/kg	i.p.	FSS	
10	Le Bourdonnec *et al*.	2008	ADL5859	-	0.3-3 mg/kg	p.o.	FSS	
11	Saitoh *et al*.	2011	KNT-127	ICR	0.1-1 mg/kg	s.c.	FSS	
12	Hudzik *et al*.	2011	AZD2327	SD	1-10 mg/kg X 4 days	p.o.	LH	

Abbreviation; FSS: Forced swim test, CSM: Conditioned suppressive motility, HE: Hyperemotionality score, LH: Learned helplessness test

## References

[1] Wittchen HU, Knäuper B, Kessler RC (1994). Lifetime risk of depression. Br. J. Psychiatry.

[2] Kessler RC, Berglund P, Demler O, Jin R, Merikangas KR, Walters EE (2005). Lifetime prevalence and age-of-onset distributions of DSM-IV disorders in the National Comorbidity Survey Replication. Arch. Gen. Psychiatry.

[3] Rush AJ, Trivedi MH, Wisniewski SR, Nierenberg AA, Stewart JW, Warden D, Niederehe G, Thase ME, Lavori PW, Lebowitz BD, McGrath PJ, Rosenbaum JF, Sackeim HA, Kupfer DJ, Luther J, Fava M (2006). Acute and longer-term outcomes in depressed outpatients requiring one or several treatment steps: a STAR*D report. Am. J. Psychiatry.

[4] Broom DC, Jutkiewicz EM, Rice KC, Traynor JR, Woods JH (2002). Behavioral effects of delta-opioid receptor agonists: potential antidepressants?. Jpn. J. Pharmacol.

[5] Jutkiewicz EM (2006). The antidepressant -like effects of delta-opioid receptor agonists. Mol. Interv.

[6] Berrocoso E, Sánchez-Blázquez P, Garzón J, Mico JA (2009). Opiates as antidepressants. Curr. Pharm. Des.

[7] Pradhan AA, Befort K, Nozaki C, Gavériaux-Ruff C,  
Kieffer BL (2011). The delta opioid receptor: an evolving target for the treatment of brain disorders. Trends. Pharmacol. Sci.

[8] Darko DF, Risch SC, Gillin JC, Golshan S (1992). Association of beta-endorphin with specific clinical symptoms of depression. Am. J. Psychiatry.

[9] Djurovic D, Milic-Askrabic J, Majkic-Singh N (1999). Serum beta-endorphin level in patients with depression on fluvoxamine. FARMACO.

[10] Emrich HM, Hollt V, Kissling W, Fischler M, Laspe H, Heinemann H, von Zerssen D, Herz A (1979). b-Endorphin-like immunoreactivity in cerebrospinal fluid and plasma of patients 
with schizophrenia and other neuropsychiatic disorders. Pharmacopsychiatry.

[11] Inturrisi CE, Alexopoulos G, Lipman R, Foley K, Rossier J (1982). ?-Endorphin immunoreactivity in the plasma of psychiatric patients receiving electroconvulsive treatment. Ann. N.Y. Acad. Sci.

[12] Kline NS, Li CH, Lehmann HE, Lajtha A, Laski E, Cooper T (1977). ?-Endorphin-induced changes in schizophrenic and depressed patients. Arch. Gen. Psychiatry.

[13] Bodkin JA, Zornberg GL, Lukas SE, Cole JO (1995). Buprenorphine treatment of refractory depression. J. Clin. Psychopharmacol.

[14] Stoll AL, Rueter S (1999). Treatment augmentation with opiates in severe and refractory major depression. Am. J. Psychiatry.

[15] Hollister LE, Johnson K, Boukhabza D, Gillespie HK (1981). Aversive effects of naltrexone in subjects not dependent on opiates. Drug ALC. Depend.

[16] Evans CJ, Keith DE, Morrison H, Magendzo K, Edwards RH (1992). Cloning of a delta opioid receptor by functional expression. Science.

[17] Kieffer BL, Befort K, Gaveriaux-Ruff C, Hirth CG (1992). The delta-opioid receptor: isolation of a cDNA by expression cloning and pharmacological characterization. Proc. Natl. Acad. Sci. U.S.A.

[18] Le Merrer J, Becker JA, Befort K, Kieffer BL (2009). Reward processing by the opioid system in the brain. Physiol. Rev.

[19] Madar I, Lever JR, Kinter CM, Scheffel U, Ravert HT, Musachio JL, Mathews WB, Dannals RF, Frost JJ (1996). Imaging of delta opioid receptors in human brain by N1-([11C]methyl) naltrindole and PET. Synapse.

[20] Porsolt RD, Le Pichon M, Jalfre M (1977). Depression: a new animal model sensitive to antidepressant treatments. Nature.

[21] Cryan JF, Markou A, Lucki I (2002). Assessing antidepressant activity in rodents: recent developments and future needs. Trends Pharmacol. Sci.

[22] Cryan JF, Valentino RJ, Lucki I (2005). Assessing substrates underlying the behavioral effects of antidepressants using the modified rat forced swimming test. Neurosci. Biobehav. Rev.

[23] Broom DC, Jutkiewicz EM, Folk JE, Traynor JR,  
Rice KC, Woods JH (002d). Nonpeptidic delta-opioid receptor 
agonists reduce immobility in the forced swim assay in rats. Neuropsychopharmacology.

[24] Jutkiewicz EM, Rice KC, Woods JH, Winsauer PJ (2003). Effects of the delta-opioid receptor agonist SNC80 on learning relative to its antidepressant-like effects in rats. Behav. Pharmacol.

[25] Detke MJ, Rickels M, Lucki I (1995). Active behaviors in the rat forced swimming test differentially produced by serotonergic and noradrenergic antidepressants. Psychopharmacology (Berl.).

[26] Rénéric JP, Lucki I (1998). Antidepressant behavioral effects by dual inhibition of monoamine reuptake in the rat forced swimming test. Psychopharmacology (Berl.).

[27] Barros HM T, Ferigolo M (1998). Ethopharmacology of imipramine in the forced-swimming test: gender differences. Neurosci. Biobehav. Rev.

[28] Rénéric JP, Bouvard M, Stinus L (2002). In the rat forced 
swimming test, chronic but not subacute administration of 
dual 5-HT/NA antidepressant treatments may produce greater effects than selective drugs. Behav. Brain Res.

[29] Nagase H, Tanaka T, Saitoh A (2002). Therapeutics of preventive medicines for mood disorders or anxiety disorders. U.S. Patent WO/2002/002117.

[30] Saitoh A, Kimura Y, Suzuki T, Kawai K, Nagase H, Kamei J (2004). Potential anxiolytic and antidepressant-like activities of SNC80, a selective delta-opioid agonist, in behavioral models in rodents. J. Pharmacol. Sci.

[31] Nagase H, Nemoto T, Matsubara A, Saito M, Yamamoto N, Osa Y, Hirayama S, Nakajima M, Nakao K, Mochizuki H, Fujii H (2010). Design and synthesis of KNT-127, a delta-opioid receptor agonist effective by systemic administration. Bioorg. Med. Chem. Lett.

[32] Nagase H, Osa Y, Nemoto T, Fujii H, Imai M, Nakamura T, Kanemasa T, Kato A, Gouda H, Hirono S (2009). Design and synthesis of novel delta opioid receptor agonists and their pharmacologies. Bioorg. Med. Chem. Lett.

[33] Kameyama T, Nagasaka M, Yamada K (1985). Effects of antidepressant drugs on a quickly-learned conditioned-suppression response in mice. Neuropharmacology.

[34] Inoue T, Tsuchiya K, Koyama T (1996). Serotonergic activation reduces defensive freezing in the conditioned fear paradigm. Pharmacol. Biochem. Behav.

[35] Miyamoto J, Tsuji M, Takeda H, Nawa H, Matsumiya T (2000). Pretreatment with diazepam suppresses the reduction in defensive freezing behavior induced by fluvoxamine in the conditioned fear stress paradigm in mice. Eur. J. Pharmacol.

[36] Kameyama T, Nagasaka M (1982). Effects of apomorphine and diazepam on a quickly learned conditioned suppression in rats. Pharmacol. Biochem. Behav.

[37] Fanselow MS, Helmstetter FJ (1988). Conditional analgesia, defensive freezing, and benzodiazepines. Behav. Neurosci.

[38] Naidu PS, Lichtman AH, Archer CC, May EL, Harris  
LS, Aceto MD (2007). NIH 11082 produces anti-depressant-like activity in the mouse tail-suspension test through a delta-opioid receptor mechanism of action. Eur. J. Pharmacol.

[39] Vergura R, Balboni G, Spagnolo B, Gavioli E, Lambert DG, McDonald J, Trapella C, Lazarus LH, Regoli D, Guerrini R, Salvadori S, Caló G (2008). Anxiolytic- and antidepressant-like activities of H-Dmt-Tic-NH-CH(CH2-COOH)-Bid (UFP-512): a novel selective delta opioid receptor agonist. Peptides.

[40] Le Bourdonnec B, Windh RT, Ajello CW, Leister LK, Gu M, Chu GH, Tuthill PA, Barker WM, Koblish M, Wiant DD, Graczyk TM, Belanger S, Cassel JA, Feschenko MS, Brogdon BL, Smith SA, Christ DD, Derelanko MJ, Kutz S, Little PJ, DeHaven RN, DeHaven-Hudkins DL, Dolle RE (2008). Potent, orally bioavailable delta opioid receptor agonists for the treatment of pain: discovery of N,N-diethyl-4-(5-hydroxyspiro [chromene-2,4-piperidine]-4-yl)benzamide (ADL5859). J. Med. Chem.

[41] Hudzik TJ, Maciag C, Smith MA, Caccese R, Pietras MR, Bui KH, Coupal M, Adam L, Payza K, Griffin A, Smagin G, Song D, Swedberg MD, Brown W (2011). Preclinical pharmacology of AZD2327—a highly selective agonist of the d opioid receptor. J. Pharmacol. Exp. Ther.

[42] Kelly JP, Wrynn AS, Leonard BE (1997). The olfactory bulbectomized rat as a model of depression: an update. Pharmacol. Ther.

[43] Song C, Leonard BE (2005). The olfactory bulbectomised rat as a model of depression. Neurosci. Biobehav. Rev.

[44] Okuyama S, Chaki S, Kawashima N, Suzuki Y, Ogawa S, Nakazato A, Kumagai T, Okubo T, Tomisawa K (1999). Receptor binding, behavioral, and electrophysiological profiles of nonpeptide corticotropin-releasing factor subtype 1 receptor antagonists CRA1000 and CRA1001. J. Pharmacol. Exp. Ther.

[45] Redmond AM, Kelly JP, Leonard BE (1997). Behavioural and neurochemical effects of dizocilpine in the olfactory bulbectomized rat model of depression. Pharmacol. Biochem. Behav.

[46] Jaako-Movits K, Zharkovsky T, Pedersen M, Zharkovsky A (2006). Decreased hippocampal neurogenesis following olfactory bulbectomy is reversed by repeated citalopram administration. Cell Mol. Neurobiol.

[47] Keilhoff G, Becker A, Grecksch G, Bernstein HG, Wolf G (2006). Cell proliferation is influenced by bulbectomy and normalized by imipramine treatment in a region-specific manner. Neuropsychopharmacology.

[48] Cryan JF, McGrath C, Leonard BE, Norman TR (1999). Onset of the effects of the 5-HT1A antagonist, WAY-100635, alone, and in combination with paroxetine, on olfactory bulbectomy and 8-OH-DPAT-induced changes in the rat. Pharmacol. Biochem. Behav.

[49] Pandey DK, Mahesh R, Kumar AA, Rao VS, Arjun M, Rajkumar R (2010). A novel 5-HT(2A) receptor antagonist exhibits antidepressant-like effects in a battery of rodent behavioural assays: approaching early-onset antidepressants. Pharmacol. Biochem. Behav.

[50] Takahashi K, Murasawa H, Yamaguchi K, Yamada M, Nakatani A, Yoshida M, Iwai T, Inagaki M, Yamada M, Saitoh A (2011). Riluzole rapidly attenuates hyperemotional responses in olfactory bulbectomized rats, an animal model of depression. Behav. Brain Res.

[51] Saitoh A, Yamaguchi K, Tatsumi Y, Murasawa H, Nakatani A, Hirose N, Yamada M, Yamada M, Kamei J (2007). Effects of milnacipran and fluvoxamine on hyperemotional behaviors and the loss of tryptophan hydroxylase-positive cells in olfactory bulbectomized rats. Psychopharmacology (Berl.).

[52] Saitoh A, Yamada M, Takahashi K, Yamaguchi K, Murasawa H, Nakatani A, Tatsumi Y, Hirose N, Kamei J (2008). Antidepressant-like effects of the delta-opioid receptor agonist SNC80 ([(+)-4-[(alphaR)-alpha-[(2S,5R)-2,5-dimethyl-4-(2-propenyl)-1- piperazinyl]-(3-methoxyphenyl)methyl]-N,N-diethylbenzamide) in an olfactory bulbectomized rat model. Brain Res.

[53] Takahashi K, Saitoh A, Yamada M, Maruyama Y, Hirose N, Kamei J, Yamada M (2008). Gene expression profiling reveals complex changes in the olfactory bulbectomy model of depression after chronic treatment with antidepressants. J. Pharmacol. Sci.

[54] Torregrossa MM, Folk JE, Rice KC, Watson SJ, Woods JH (2005). Chronic administration of the delta opioid receptor agonist (+)BW373U86 and antidepressants on behavior in the forced swim test and BDNF mRNA expression in rats. Psychopharmacology.

[55] Watanabe A, Tohyama Y, Nguyen KQ, Hasegawa S, Debonnel G, Diksic M (2003). Regional brain serotonin synthesis is increased in the olfactory bulbectomy rat model of depression: an autoradiographic study. J. Neurochem.

[56] Hasegawa S, Watanabe A, Nguyen KQ, Debonnel G, Diksic M (2005). Chronic administration of citalopram in olfactory bulbectomy rats restores brain 5-HT synthesis rates: an autoradiographic study. Psychopharmacology (Berl.).

[57] Jenny M, Winkler C, Spetea M, Schennach H, Schmidhammer H, Fuchs D (2008). Non-peptidic delta-opioid receptor antagonists suppress mitogen-induced tryptophan degradation in peripheral blood mononuclear cells *in vitro*. Immunol. Lett.

[58] Torregrossa MM, Isgor C, Folk JE, Rice KC, Watson SJ, Woods JH (2004). The delta-opioid receptor agonist (+) BW373U86 regulates BDNF mRNA expression in rats. Neuropsychopharmacology.

[59] Torregrossa MM, Jutkiewicz EM, Mosberg HI, Balboni G, Watson SJ, Woods JH (2006). Peptidic delta opioid receptor agonists produce antidepressant-like effects in the forced swim test and regulate BDNF mRNA expression in rats. Brain Res.

[60] Siuciak JA, Clark MS, Rind HB, Whittemore SR, Russo AF (1998). BDNF induction of tryptophan hydroxylase mRNA levels in the rat brain. J Neurosci Res.

[61] Narita M, Kuzumaki N, Miyatake M, Sato F, Wachi H, Seyama Y, Suzuki T (2006). Role of delta-opioid receptor function in neurogenesis and neuroprotection. J. Neurochem.

[62] Saitoh A, Sugiyama A, Nemoto T, Fujii H, Wada K, Oka J, Nagase H, Yamada M (2011). The novel δ opioid receptor agonist KNT-127 produces antidepressant-like and antinociceptive effects in mice without producing convulsions. Behav. Brain Res.

[63] Tortella FC, Echevarria E, Robles L, Mosberg HI, Holaday JW (1988). Anticonvulsant effects of mu (DAGO) and delta (DPDPE) enkephalins in rats. Peptides.

[64] Comer SD, Hoenicke EM, Sable AI, McNutt RW, Chang K-J, De Costa BR, Mosberg HI, Woods JH (1993). Convulsive effects of systemic administration of the delta opioid agonist BW373U86 in mice. J. Pharmacol. Exp. Ther.

[65] Broom DC, Jutkiewicz EM, Folk JE, Traynor JR, Rice KC, Woods JH (2002). Convulsant activity of a non-peptidic delta-opioid receptor agonist is not required for its antidepressant-like effects in Sprague-Dawley rats. Psychopharmacology (Berl.).

[66] Aceto MD, May EL, Harris LS, Bowman ER, Cook CD (2007). Pharmacological studies with a nonpeptidic, delta-opioid (-)-(1R,5R,9R)-5,9-dimethyl-2'-hydroxy-2-(6-hydroxyhexyl)-6,7-benzomorphanhydrochloride ((-)-NIH 11082). Eur. J. Pharmacol.

[67] Petrillo P, Angelici O, Bingham S, Ficalora G, Garnier M, Zaratin PF, Petrone G, Pozzi O, Sbacchi M, Stean TO, Upton N, Dondio GM, Scheideler MA (2003). Evidence for a selective role of the delta-opioid agonist [8R-(4bS*,8aalpha,8abeta,12bbeta)] 7,10-Dimethyl-1-methoxy-11-(2-methylpropyl)oxycarbonyl5,6,7,8, 12,12b-hexahydro-(9H)-4,8-methanobenzofuro[3,2-e]pyrrolo[2,3-g]isoquinoline hydrochloride (SB-235863) in blocking hyperalgesia associated with inflammatory and neuropathic pain responses. J. Pharmacol. Exp. Ther.

[68] Codd EE, Carson JR, Colburn RW, Stone DJ, Van Besien CR, Zhang SP, Wade PR, Gallantine EL, Meert TF, Molino L, Pullan S, Razler CM, Dax SL, Flores CM (2009). JNJ-
20788560 [9-(8-azabicyclo[3.2.1]oct-3-ylidene)-9H-xanthene-3-
carboxylic acid diethylamide], a selective delta opioid receptor
agonist, is a potent and efficacious antihyperalgesic agent that does
not produce respiratory depression, pharmacologic tolerance,
or physical dependence. J. Pharmacol. Exp. Ther.

[69] Le Bourdonnec B, Windh RT, Leister LK, Zhou QJ, Ajello CW, Gu M, Chu GH, Tuthill PA, Barker WM, Koblish M, Wiant DD, Graczyk TM, Belanger S, Cassel JA, Feschenko MS, Brogdon BL, Smith SA, Derelanko MJ, Kutz S, Little PJ, DeHaven RN, DeHaven-Hudkins DL, Dolle RE (2009). Spirocyclic delta opioid receptor agonists for the treatment of pain: discovery of N,N-diethyl-3-hydroxy-4-(spiro[chromene-2,4-piperidine]-4-yl) benzamide (ADL5747). J. Med. Chem.

[70] Portoghese PS (1989). Bivalent ligands and the message-address concept in the design of selective opioid receptor antagonists. Trends Pharmacol. Sci.

[71] Pradhan AA, Becker JA, Scherrer G, Tryoen-Toth P, Filliol D, Matifas A, Massotte D, Gavériaux-Ruff C, Kieffer BL (2009). *In vivo* delta opioid receptor internalization controls behavioral effects of agonists. PLoS One.

